# An exploratory assessment of the applicability of direct-to-consumer genetic testing to translational research in Japan

**DOI:** 10.1186/s13104-021-05696-4

**Published:** 2021-07-23

**Authors:** Masahiro Inoue, Shota Arichi, Tsuyoshi Hachiya, Anna Ohtera, Seok-Won Kim, Eric Yu, Masatoshi Nishimura, Kazuhito Shiosakai, Takeshi Ohira

**Affiliations:** 1 HealthData Lab, Yahoo! Japan Corporation, Kioi Tower, Tokyo Garden Terrace Kioicho, 1-3, Kioi-cho, Chiyoda-ku, Tokyo, 102-8282 Japan; 2 Real World Evidence Solutions, IQVIA Solutions Japan K.K, Takanawa 4-10-18, Minato-ku, Tokyo, 108-0074 Japan; 3 Daiichi Sankyo Co. Ltd, Tokyo, Japan

**Keywords:** DTC, SNPs, Phenotype, PheWAS, Translational research, GRIN2B

## Abstract

**Objective:**

In order to assess the applicability of a direct-to-consumer (DTC) genetic testing to translational research for obtaining new knowledge on relationships between drug target genes and diseases, we examined possibility of these data by associating SNPs and disease related phenotype information collected from healthy individuals.

**Results:**

A total of 12,598 saliva samples were collected from the customers of commercial service for SNPs analysis and web survey were conducted to collect phenotype information. The collected dataset revealed similarity to the Japanese data but distinguished differences to other populations of all dataset of the 1000 Genomes Project. After confirmation of a well-known relationship between *ALDH2* and alcohol-sensitivity, Phenome-Wide Association Study (PheWAS) was performed to find association between pre-selected drug target genes and all the phenotypes. Association was found between *GRIN2B* and multiple phenotypes related to depression, which is considered reliable based on previous reports on the biological function of GRIN2B protein and its relationship with depression. These results suggest possibility of using SNPs and phenotype information collected from healthy individuals as a translational research tool for drug discovery to find relationship between a gene and a disease if it is possible to extract individuals in pre-disease states by properly designed questionnaire.

**Supplementary Information:**

The online version contains supplementary material available at 10.1186/s13104-021-05696-4.

## Introduction

Clinical trials conducted by pharmaceutical companies are sometimes discontinued due to inability to reproduce treatment effects previously confirmed in animal studies. One possible reason is the difference in drug response between disease model animals and humans. Recent advances have led to a method to evaluate similarities in drug response between animals and humans based on gene expression profiles [1]. However, it does not completely address species difference between animals and humans. Therefore, the importance of translational research is recognized in which the relationship between a target gene and a disease is validated using data obtained from humans [2]. Probably the most straightforward approach is to obtain patient samples and examine the expression of the target gene or protein in a disease of interest; however, this cannot easily be performed. On the other hand, the Genome-Wide Association Study (GWAS) method could estimate the association between a target gene and a disease without taking patient samples by comprehensively searching for single-nucleotide polymorphism (SNP) which differs in frequency between patients and healthy individuals. However, it may be challenging to collect a sufficient number of samples.

A previous study introduced the association between customer survey data and genotypes using the direct-to-consumer (DTC) genetic testing for antidepressant efficacy [3]. Also, in the area of personalized precision medicine, the DTC genetic testing for big data is expected to develop new drugs [4]. Therefore, to assess the applicability of a DTC genetic testing to translational research for obtaining new knowledge on relationships between drug target genes and diseases in this study, we examined the possibility of DTC genetic testing by the relationship between a target gene and a disease using a panel-based web survey of healthy individuals. SNPs of the target gene were associated with a disease via various phenotypic information related to the disease. A set of target genes were selected in advance and the Phenome-Wide Association Study (PheWAS) was employed instead of the GWAS to perform a hypothesis-free cross-phenotype search [5].

## Main text

### Methods

This study was performed as shown in Additional file 1: Figure S1. All samples were supplied by participants extracted from the customers of Japanese Direct-to-Consumer (DTC) genetic testing service, HealthData Lab (Yahoo! Japan Corporation, Tokyo, Japan), with consent of opt-out. In total 12,596 subjects were approached and 2 subjects (0.016%) did not opt-out. All individuals were irreversibly anonymized for confidentiality. Two genotyping platforms were used at RIKEN GENESIS (Tokyo, Japan). Sample QC was performed both individual and SNP levels. A mandatory questionnaire was conducted on all the participants on the HealthData Lab website for testing individual phenotypes. The questionnaire consists of one-hundred sixty-one questions as shown in Additional file 1: Table 1. These questions were designed to collect comprehensive information of the participants related to lifestyle, taste preference, health checkup summary, medical history and mental health condition, and not specifically designed to collect disease related information. These data were used to divide participants into case and control groups, thus generating various phenotypes, based on the answer and categorization method with multiple thresholds. 447 phenotypes with case number > 100 was considered reliable and used for analysis. Using these phenotypes, association between *ALDH2* and alcohol sensitivity was tested as a positive control to assess the reliability of the data set and analysis method while other pre-selected five genes was used as negative controls. *ALDH2* has been known as a gene which is related to alcohol metabolism [6]. According to STRING database [7], a couple of genes belonging to an alternative metabolic pathway are shown to have relationship with *ALDH2* but the degree of association between these genes and alcohol metabolism is much lower compared to *ALDH2* [6]. Therefore, *ALDH2* was used as a single and definite positive control. In this analysis, threshold of minor allele frequency was 0.03, which was used in the previous study related to neuroscience research [8]. These genes were/are targets of drugs development project in DaiichiSankyo Co., Ltd. for various disease areas including central nervous system diseases, ophthalmic diseases, immunological diseases, dyslipidemia. Then, Phenome-wide association study (PheWAS) was performed to test association between SNPs belonging to these drug target genes and all the 447 phenotypes. For these association analyses, logistic regression using an additive genetic model was employed for the following three cohorts, Male, Female, All (Male and Female). All method details are explained in the Additional file.

### Results

We performed a principal component analysis (PCA) and investigated characteristics of this study data compared to currently published dataset. As shown in Additional file1: Figure S3, our dataset revealed similarity to the Japanese data in the 1000 Genomes Project dataset, but distinguished differences to other populations of all and East Asian dataset of the 1000 Genomes Project dataset.

We compared the population of each prefecture with the Ministry of Internal Affairs and Communications (MIC) survey [9] to confirm the similarity between the population ratio used in this study and the national population. Additional file 1: Figure S4 indicates population ratio of data used in this study matched the general population investigated by the national survey.

**Fig. 1:**
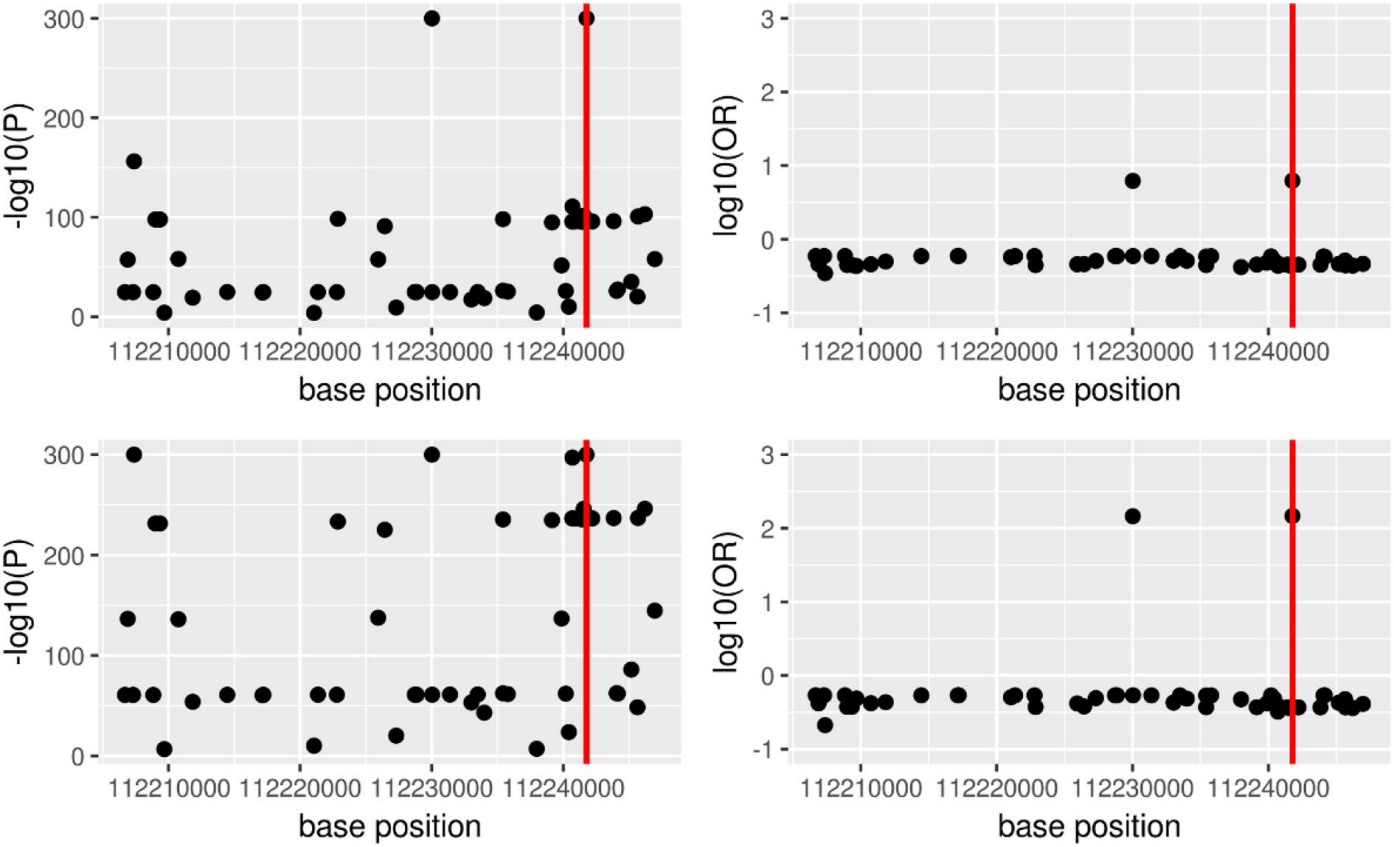
Association with *ALDH2* and alcohol sensitivity obtained from the All cohort. rs671 is indicated by a red line. Upper part: alcohol intake frequency, Lower part: alcohol flush reaction, Left part: -log10 (P), Right part: log10 (OR) where P represents p-value while OR represents Odds ratio

In the examination of a relationship between *ALDH2* and alcohol-sensitive phenotypes as positive control, a significant association was found between SNPs including previously reported rs671 and multiple SNPs located around it and alcohol sensitivity represented as drinking frequency and flush reaction [10]. Figure 1 shows the result obtained from All cohort as a representative case. No significant difference was found in terms of p-value and OR between the All cohort and Male /Female cohorts. Odds ratio was higher for the flushing response than for drinking frequency. Only rs671 and rs4646776 showed very high values (147.0 and 145.6) and all the other SNPs showed values around 1 for the flush reaction. On the other hand, in all the five genes selected as drug target and used as negative controls, no p-value smaller than 1.1 × 10^–7^, which is the Bonferroni adjusted threshold corresponding to p-value = 0.05, was obtained from association analysis for both alcohol drinking frequency and flush reaction.

**Fig. 2:**
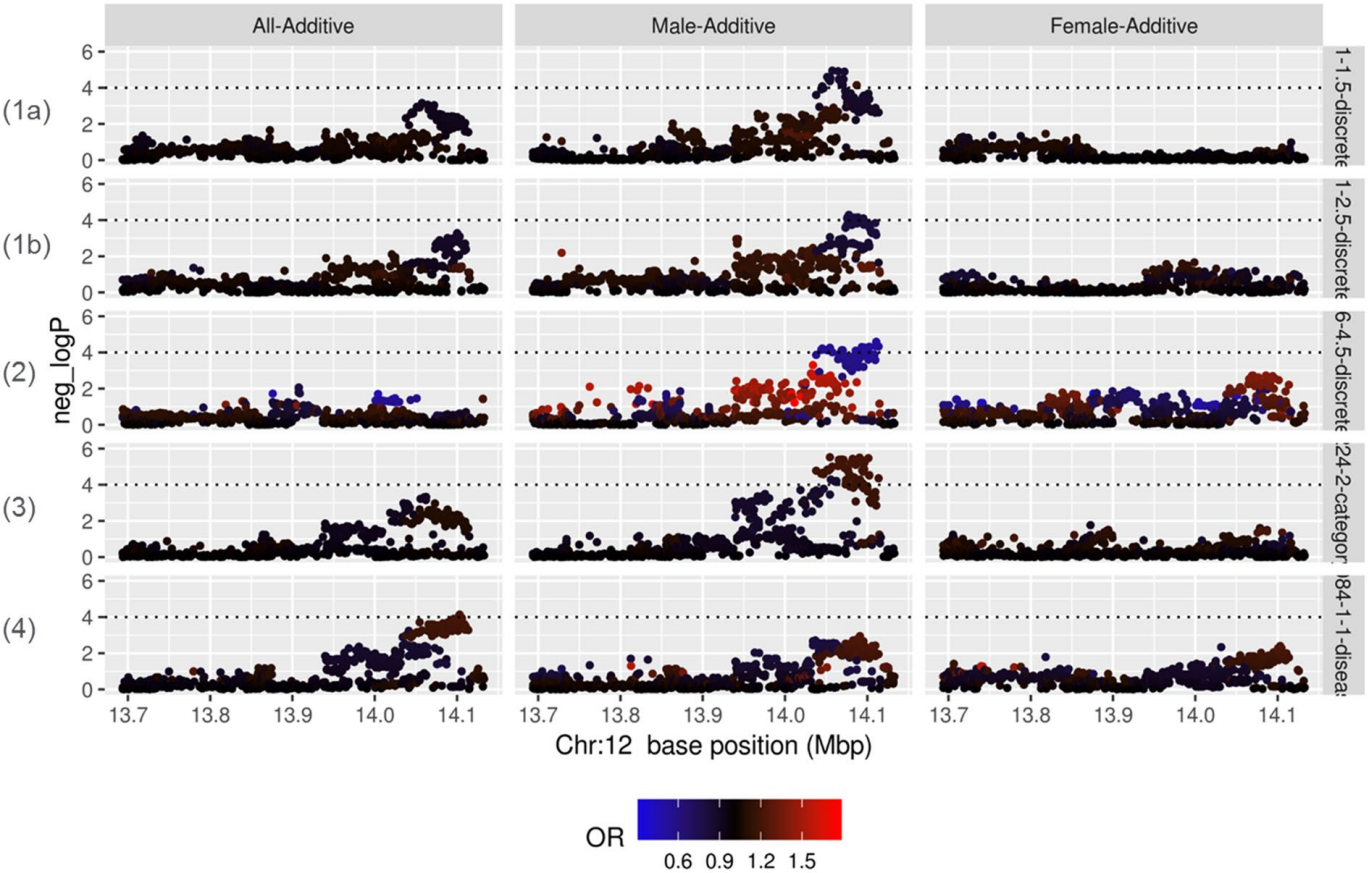
Plot of p-values and OR for all the combinations of phenotypes and cohorts listed in Table. 1. The color of each plot represents OR. The horizontal dotted line corresponds to the threshold p-value of 1 × 10^–4^. Case and control definition shown as (1a), (1b), (2), (3), and (4) is the same as Table 1

Among all associations between all the SNPs corresponding to the five drug target genes and all the phenotypes, no phenotype was found with p-value smaller than 1.1 × 10^–7^ for each model. Since all the SNPs are not mutually independent and all the phenotypes are not mutually independent either, requirement for the phenotypes to be considered significant was changed to p-value < 1 × 10^–4^, corresponding to FDR = 0.509, to generate a manageable number of phenotypes for manual investigation while keeping the ratio of false positive about 50%. As expected, even with this p-value, the number of associations was quite limited. Four genes out of five genes had a very limited number (zero or one) of association with the phenotypes which seem disease related and hence not considered reliable for further investigation. However, one of the genes (*GRIN2B*) had an association with a total of 22 phenotypes (Additional file 2: Table 5). As the major phenotype among these phenotypes, four of them are related to depression as shown in Table 1 (left part) and considered reliable enough for further investigation. Table 1 (right part) shows the number of case and control for all the cohorts (All, Male, Female) and the depression related phenotypes. It was found that the significant association was observed mostly in Male cohort and not observed in Female cohort. This male specific association is also obvious in Fig. 2, which shows the plot of p-value and OR along the chromosome for the phenotypes and cohorts corresponding to Table 1 (right part).

**Table 1:** Significant association observed between *GRIN2B* and phenotypes related to depression for certain cohorts (left part) and the number of case and control for all the cohorts (All, Male, Female) (right part). The cells enclosed by a thick line correspond to the cohort shown in the left part for which significant association is found

No	Question	Case (Definition)	Control (Definition)	Cohort	No. of associated SNPs	SNPs with the lowest p-value	P-value	OR	All Cohort		Male Cohort		Female Cohort	
									Case (N)	Control (N)	Case (N)	Control (N)	Case (N)	Control (N)
1a	Did you feel nervous?	Always Often Sometimes Rarely	Never	Male	22	rs11055680	1.13 × 10^–5^	0.853 (0.794–0.916)	5,849	6,795	2,626	4,072	3,214	2,723
b	Did you feel nervous?	Always Often Sometimes	Rarely Never	Male	12	rs10845862	5.14 × 10^–5^	0.801 (0.719–0.891)	2,106	10,538	897	5,801	1,200	4,737
2	Did you feel you were a worthless person?	Always	Often Sometimes Rarely Never	Male	23	rs7970022	2.62 × 10^–5^	0.493 (0.355–0.686)	387	12,257	158	6,540	220	5,717
3	Did you feel uninterested in the things or could not really enjoy the things for the past one month?	Yes	No	Male	65	rs759619	8.51 × 10^–7^	1.21 (1.12–1.31)	3,819	8,825	1,948	4,747	1,871	4,066
4	Did you have a history of depression?	Yes	No	All	2	rs4763360	7.34 × 10^–5^	1.23 (1.11–1.35)	1,061	11,583	545	6,153	516	5,421

## Discussion

Data generated by this study demonstrated similarity to Japanese genome dataset in the 1000 Genomes Project and regional characteristics compared to the national survey. This indicates that these data may be applied further to Japanese PheWAS studies with additional questions.

We examined the reliability of association between SNPs and the phenotype information collected by web-based survey using a known relationship as a positive control. The SNP, rs671 is known to produce an amino acid mutation (E504K) along with a nucleic acid mutation (1510G > A) to reduce its acetaldehyde metabolizing ability [10]. The strong association observed in this study between rs671 and alcohol sensitivity was consistent with this known relationship. In addition to rs671, an association between the SNP, rs4646776 and alcohol sensitivity was also observed possibly because rs4646776 is a tag SNP of rs671 [11]. Associations between these SNPs and flushing reaction was stronger than associations between these SNPs and the drinking frequency. This result is interpreted to be appropriate as the flushing reaction is considered to reflect alcohol sensitivity more directly than the drinking frequency. Furthermore, the associations with alcohol sensitivity were observed only for the SNPs corresponding to *ALDH2* and not observed for the SNPs corresponding to the five drug target genes as negative controls. From the above, collection of reliable SNPs information and phenotype information is considered possible through web questionnaire if questionnaire is properly designed to define a phenotype of interest.

We conducted PheWAS cross-phenotype searches for the five drug target genes in a hypothesis-free manner and found association between *GRIN2B* and multiple depression-related phenotypes. This result appeared to be reliable, as GRIN2B protein mediates excitatory neurotransmission in the brain as a subunit of the NMDA receptor and there were already several previous studies about the association between *GRIN2B* and depression [12, 13]. rs1805502 was found associated with treatment-resistant depression in Han Chinese population [12], while rs220549 was found associated with neuroticism, which is an endophenotype of MDD in European population [13]. In the present study, no association was found between these two SNPs and depression related phenotypes, which may be because of the difference of ethnicity. Furthermore, it was found that the significant association was observed mostly in Male cohort and not observed in Female cohort. The difference between the sexes in terms of association with depression-related phenotypes might be attributed to the difference in the causes of depressive mood. In female, hormonal state changes are known to have substantial impact on mood such as postpartum and postmenopausal depressions [14]. On the other hand, depressive mood caused by hormonal changes is less frequent in male [15]. The association between *GRIN2B* and depressive phenotypes was not detected in female potentially because hormonal changes might have a larger impact on depressions in female than *GRIN2B*, which might be involved in depression by a different mechanism. It may be possible to prove this hypothesis by confirming if association is found between *GRIN2B* genotype and depression related phenotypes using female subpopulation in which hormonal state is considered stable. However, the questions used in this study were designed for gathering comprehensive information of the participants and specific questions to estimate the hormonal state change elicited during postpartum and postmenopausal period of female participants were not included. Therefore, further analysis using a newly designed set of questions to estimate female hormonal state is necessary to test the hypothesis.

In contrast, we identified a very limited disease associations in the other four genes. This is consistent with the original expectation because the questionnaire was not designed to collect disease related information, especially the target disease of these four genes (ophthalmic diseases, immunological diseases, dyslipidemia), and the number of participants were small. Therefore, it is conceivable that the association between *GRIN2B* and depression related phenotypes were obtained because the web survey contained multiple questions relating to depression-related mental states. Please note that this association was not obtained from standard case/control study where case is usually defined as patients having certain disease but from the data obtained from a larger number of healthy individuals before being diagnosed as the disease. In addition, while the number of SNPs was high for *GRIN2B* (784), the number of SNPs were medium or low for other four genes (184, 43, 1, and 1) which may worked favorably for *GRIN2B* compared to other genes in terms of detection of association. Since the relationship between *GRIN2B* and depression is much more complicated than the relationship between *ALDH2* and alcohol sensitivity, this type of association analysis could be applied to find association between a gene and a disease of some complexity with involvement of multiple genes and environmental factors when the above conditions regarding questionnaire are met.

Based on the above observation, although this study was conducted in a retrospective and hypothesis-free manner using already obtained data, it makes more sense to proactively collect phenotype data to test or refine a hypothesis between a gene and a disease as described above in the case of *GRIN2B* and depression by eliminating the effect of hormonal state change. In general, collection of phenotype information to test a hypothesis is feasible if it is possible 1) to define multiple pre-disease phenotypes of the target disease, 2) to collect these pre-disease phenotype information using a web-based questionnaire, and 3) to obtain a large number of cases. If such scheme would be implemented, analyzing SNPs and phenotype information using data obtained by DTC genetic testing and web-based questionnaire could be an effective translational research tool for drug discovery.

### Conclusion

Using SNPs and phenotype information obtained from healthy individuals, well documented relationships between *ALDH2* and alcohol-sensitivity were confirmed and reproduced. Furthermore, associations between *GRIN2B* and multiple phenotypes related to depression were found. These results demonstrate the possibility of using DTC genetic testing service as a translational research tool for drug discovery to find relationship between a gene and a disease if it is possible to extract individuals in pre-disease states by properly designed questionnaire.

## Limitation

This study has a limitation. We performed all analyses using commercial dataset with end of service. Furthermore, this dataset was used under the terms of use that all dataset cannot be public disclosure following the genome guideline, and all customers agreed on this terms of use; therefore, we cannot publicly provide dataset for readers to confirm reproducibility or any further information. Furthermore, we cannot disclose the name of genes except *GRIN2B* since drug development projects targeting these genes are still active.

## Supplementary Information

**Additional file 1: **Method details for this study and supplementary Table 1, 2, 3 and 4, and supporting figure S1, S2, S3 and S4.

**Additional file 2:** Full list of the number of associated SNP of GRIN2B for 447 phenotypes.

## Data Availability

This study used commercial dataset with end of service; therefore, we cannot publicly provide dataset for readers to confirm reproducibility or any further information because all raw data has been deleted. However, de-identified analysis data could be provided with reasonable scientific reason. Please contact corresponding author directly.

## Abbreviations

DTC: Direct-to-Consumer
FDR: False Discovery Rate
GWAS: Genome-Wide Association Study
MIC: Ministry of Internal Affairs and Communications
PheWAS: Phenome Wide Association Study
PCA: Principal Component Analysis
QC: Quality Control
SNP: Single Nucleotide Polymorphism
