# Supplementary material for: An exploratory assessment of the applicability of direct-to-consumer genetic testing to translational research in Japan
Source: BMC Res Notes. 2021 Jul 23;14:282. doi: 10.1186/s13104-021-05696-4 (PMC8305957; doi:10.1186/s13104-021-05696-4)
Supplement: Supplementary file 1 — Additional file 1: Method details for this study and supplementary Table 1, 2, 3 and 4, and supporting figure S1, S2, S3 and S4. [file 13104_2021_5696_MOESM1_ESM.docx]

An exploratory assessment of the applicability of direct-to-consumer genetic testing to translational research in Japan

Masahiro Inoue^1^, Shota Arichi^1^, Tsuyoshi Hachiya^1^, Anna Ohtera^2^, Seok-Won Kim^2^, Eric Yu^2^, Masatoshi Nishimura^3^, Kazuhito Shiosakai^3^, Takeshi Ohira^3*^

^1^HealthData Lab, Yahoo! Japan Corporation, Kioi Tower, Tokyo Garden Terrace Kioicho, 1-3, Kioi-cho, Chiyoda-ku, Tokyo, 102-8282, Japan

^2^Real World Evidence Solutions, IQVIA Solutions Japan K.K, Takanawa 4-10-8, Minato-ku, Tokyo, 108-0074, Japan

^3^Daiichi Sankyo Co. Ltd., Japan

*E-mail: ohira.takeshi.cy@daiichisankyo.co.jp

Materials and Methods

2 Genomic DNA samples

All samples were supplied by participants extracted from the customers of Japanese Direct-to-Consumer (DTC) genetic testing service, HealthData Lab (Yahoo! Japan Corporation, Tokyo, Japan). The participants purchased or were provided with the genetic testing kit for DTC generic testing service via the internet and registered their Yahoo! JAPAN ID and the code of the kit on the web page for this service. Consent was obtained from the participants using the enclosed form within the kit where they consented for their data to be used for research. Participants collected their saliva using a saliva collection kit, Oragene®·DNA (OG-500) (DNA Genotek, Ottawa, Canada). The saliva samples collected were sent with signed consent forms to the HealthData Lab and the analysis results were provided to the participants through the HealthData Lab website. DNA for genotyping assay was extracted from the saliva samples in accordance with the manufacturer’s instructions. Measurements of degeneration and concentration of the obtained genomic DNA were performed by gel electrophoresis and fluorescence-based dsDNA assay (PicoGreen® dsDNA assay kit; Thermo Fisher Scientific, Waltham, MA), respectively. Where genomic DNA was degenerated or its concentration was low, we sent a new saliva collection kit to the participants and requested additional saliva collection. Additional consent was obtained from subjects by offering an opt-out through the web site (Figure S2).

3 Genotyping

Two genotyping platforms, the Illumina HumanCore-12 Custom BeadChip v1.0 and HumanCore-24 Custom BeadChip v1.2 (Illumina, San Diego, CA) were used. The Illumina HumanCore-12 Custom BeadChip contained 302,072 markers, including approximately 2,000 selected markers. The Illumina HumanCore-24 Custom BeadChip contained 309,725 markers, including approximately 3,000 selected markers. When the call rate (percentage of SNPs successfully genotyped by sample) for ordinal SNPs, contained in the Illumina HumanCore platform, was under 85 %, we sent a new saliva collection kit to the customers and re-genotyped.

4 Sample QC

4.1 Individual level

Sample quality control (QC) was conducted at individual levels, which are missing genotype data, gender conformity, ethnicity and kinship.

To exclude individuals who are missing genotype data, subjects with local call rates (<0.99) were removed.

For gender conformity, samples from the subjects with inconsistent sex information between X chromosomal SNP genotypes and the questionnaire were excluded before genotype datasets were merged.

After datasets were merged, samples whose estimated ancestry was not Japanese were excluded from the subjects by performing principal component analysis [4] with the 1000 Genomes Project reference panel (Phase 3 version 5) [5]. Additionally, either of individual pairs estimated to be related (PI_HAT > 0.1875) were also excluded.

4.2 SNPs level

Sample QC was conducted at SNPs levels. SNPs on sex chromosomes, with low call rates (<0.99), low Hardy-Weinberg equilibrium exact test p-values <1×10^-6^, or low minor allele frequencies (MAFs, <0.01) were excluded. These QC procedures were performed with PLINK (v1.9) which is whole genome data analysis toolset [6].

5 Data merge

The two data platforms contained common markers. We extracted genotyped data associated with these common markers and merged these data after “4 Sample QC”. The merged data were applied to the aforementioned “4 Sample QC” again. 12,385 subjects remained for later analyses (Supplementary Table 2).

6 Imputation

Haplotype phasing and imputation were performed using EAGLE2 (v2.4.1) [7] and Minimac3 (v2.0.1) [8], respectively, with the 1000 Genomes Project reference panel (Phase 3 version 5). *ALDH2* was selected as a positive control, and other five genes, which are the drug targets of in-house compounds, were selected. Then the SNPs within these six genes and within 100 bp upstream of these genes are used for analysis. After the imputation, SNPs with low imputation quality (R^2^ < 0.3) were excluded. 51 SNPs for ALDH2 and a total of 1,013 SNPs for the other five genes were used for analysis.

7 Questionnaires

Questionnaire items included lifestyle habits relating to alcohol intake frequency and alcohol flush reaction. These phenotypes related to alcohol sensitivity were used to confirm the association between the genotype of *ALDH2* as a positive control in this study (Supplementary Table 3). Case group was defined in two ways, “people who never drink” or “people who develops flushes”.

All questionnaire items were used to divide participants into case and control groups, according to the type of answers (Supplementary Table 4).

1-a) Unordered categorical variables with a single choice: Case was defined as selected while control was defined as non-selected for each category. The number of phenotypes is defined as 1.

1-b) Unordered categorical variables with multiple choices: Case was defined as individuals who selected while control was defined as individuals who did not select for each category. The number of phenotypes is defined as the number of choices.

2) Ordered categorical variables: Case and control were defined based on different thresholds while keeping the order. The number of phenotypes is defined as 1.

A total of 593 phenotypes were observed. By selecting phenotypes in which the whole population of the case, including male and female, is greater than 100, a total of 447 phenotypes were subjected to an association analysis.

8 Association analysis and Phenome-wide association study (PheWAS)

Alcohol sensitivity is known as one of the phenotypes defined mostly by a single gene (*ALDH2*) [9]. Therefore, association between 51 SNPs located within and 100 bp upstream of *ALDH2* and alcohol sensitivity which were defined by alcohol intake frequency and flush reaction were investigated (Supplementary Table 3).

To evaluate whether PheWAS could produce any useful information for drug discovery, we examined association between the five genes which were selected as drug targets and all of the 447 phenotypes after QC (case number > 100 for the whole population) in a hypothesis-free manner. A total of 1,013 SNPs within and 100 bp upstream of each gene (the number of SNPs for each gene is 784, 184, 43, 1, and 1, respectively) were used for analysis. Additionally, since these five genes were not considered related to alcohol sensitivity, they were used as negative controls to determine whether this association is specific to *ALDH2* or not.

As stated before, since the questionnaire was not necessarily related to disease and the number of the participants was relatively small, around 12,000, the probability of obtaining a significant association between SNPs of drug target genes and phenotypes defined by these questions was considered low. However, this study was conducted to assess limitation and future improvement of this approach with slight expectation of finding any association with phenotypes that are not intuitively related to the target diseases because such findings may lead to a novel insight and deeper understanding of the function of these genes.

Logistic regression using an additive genetic model was performed to test association between genotype of each SNP and phenotypes with all participants (*All*), and within sexes (*Male* and *Female*). For Male and Female cohorts, for each of the SNPs tested, the following equation was used for regression:

$$\log(\frac{p_{ijk}}{1-p_{ijk}})=\beta_{0, ij}+\beta_{1, ij}g_{ik}$$

where *i* represents *i*th SNP (*i*=1,…, 1,013), *j* represents *j*th phenotype (*j*=1,…, 447) , and *k* represents *k*th individual, and *g_ik_* indicates the number of minor alleles as shown below:

$$g_{ik}=\left\{ \begin{aligned} 0, major homozygotes \\ 1, heterozygotes \\ 2, minor homozygotes \end{aligned} \right.$$

$p_{ijk}$indicates the probability of a case for phenotype *j* given *g_ik_*. Note that $p_{ijk}/(1-p_{ijk})$ represents odds. Odds ratio (OR), which is defined as the ratio of odds of possessing an additional minor allele, is given by $exp(\beta_{1, ij})$. For All cohort, a slightly modified equation was used to include sex as a covariate for regression:

$$\log(\frac{p_{ijk}}{1-p_{ijk}})=\beta_{0, ij}+\beta_{1, ij} gik+\beta_{2, ij} s_{k}$$

where *s_k_* indicates a value as shown below

$$s_{k}=\left\{ \begin{aligned} 0, Male \\ 1, &Female \end{aligned} \right.$$

For each of these three cohort, p-value, OR and 95% confidence intervals were calculated for all the combinations of genotypes and phenotypes. Bonferroni correction was employed to adjust multiplicity in terms of the number of SNPs and phenotypes, generating a threshold p value of 1.1 x 10^-7^ (0.05 / (1,013 x 447)). Since no association was detected, the threshold was increased while keeping acceptable reliability by monitoring False Discovery Rate (FDR). A p value of 1 x 10^-4^, with corresponding FDR=0.509, which is not very low but considered acceptable for further investigation, was used to detect associations. All statistical analyses were performed using PLINK (v1.9) and R (v3.6.2).

Supporting Tables

**Supplementary Table 1.** The questionnaire consisted of one-hundred sixty-one questions

| No |  | Question | Choices |
| --- | --- | --- | --- |

| **1** |  | Your biological sex | Male Female |
| --- | --- | --- | --- |
| **2** |  | Have you ever smoked? | No Yes |
| **3** |  | For those who answered Yes: Do you currently smoke? | NO YES From time to time |
| **4** |  | Did anybody whom you lived with longer than 10 years smoke? | No Yes |
| **5** |  | How often do you possibly inhale the smoke of other smokers (longer than 1 hour per day) outside of your house (workplace, etc.)? | Rarely Almost every day 1-4 days a week 1-3 days a month |
| **6** |  | How much do you drink? | Never Every day 5-6 days a week 3-4 days a week 1-2 days a week 1-3 days a month Less than the above |
| **7** |  | For those who drink alcohols: Does the number of tobaccos you smoke increase while you drink? | Don't smoke More than usual As usual Less than usual |

| **8** |  | Does your face turn red soon after drinking alcohol? | Turns red If anything, turns red Don't turn red Don't know/Never drink |
| --- | --- | --- | --- |
| **9** |  | Do you experience palpitation or headache soon after drinking alcohol? | Yes If anything, yes No Don't know/Never drink |
| **10** |  | How often do you use your mobile phone? | Never Every day 1-4 days a week 1-3 days a month |
| **11** |  | For mobile phone users only: What is the phone type? (multiple answers allowed) | Feature phone Smartphone（iPhone) Smartphone（Android） Smartphone（Other） |
| **12** |  | Do you use the internet? | No Yes |
| **13** |  | Users only: What is the information you search over the internet? (multiple answers allowed) | Searching News Video Catching up Q&A Shopping Game Stock price/Economy Sports Map/Region（Shop, Sightseeing, timetable） Health/Illness Hobby Weather Recipe Fortune telling Other |
| **14** |  | How often do you eat breakfast? (Excluding those who drink coffee /tea only) per week? | Almost none Almost every day 3-5 days a week 1～2 days a week |
| **15** |  | How often you take snack or late-night snack after 10 pm at night per week? | Almost none Almost every day 3-5 days a week 1～2 days a week |
| **16** |  | How much do you eat for your ordinary meal? | Eat until I'm 80 % or less full I eat in moderation I stuff myself |
| **17** |  | How often /week do you eat the dish cooked with oil, such as stir-fried /deep fried? | Never Almost every day 3-5 days a week 1-2 days a week |
| **18** |  | Do you avoid the burnt part of cooked meat /fishes? | No Yes |
| **19** |  | Do you avoid the food possibly rotten? | No Yes |
| **20** |  | Do you try not to take too much salt? | No Yes |
| **21** |  | Do you try to avoid taking too much animal fat such as butter and bacon? | No Yes |
| **22** |  | Do you try to take Green and Yellow vegetables? | No Yes |
| **23** |  | Are you allergic to any food? | No Yes |
| **24** |  | For those who answered yes: Please specify the food you are allergic to (multiple answers allowed) | Shrimp Crab Wheat Buckwheat Egg Milk Peanuts Cashew nuts Walnuts Abalone Squid Salmon roe Salmon Mackerel Soy Sesame Beef Pork Chicken Orange Bana Kiwifruit Apple Peach Yam Matsutake mushroom gelatin Other |
| **25** |  | For those who answered yes: Do you still have food allergy? | No Yes |
| **26** |  | Does coffee prevent you from sleeping? | No Yes Don't know |
| **27** |  | Are you lactose-intolerant? | No Yes Don't know |
| **28** |  | For those who answered yes: when did it start? | From childhood Since growing up |
| **29** |  | How often do you take dietary supplement per week? | Never Almost every day 3-5 days a week 1-2 days a week |
| **30** |  | For those who take dietary supplement: Please specify (Multiple answers allowed) | Multi-Vitamin Vitamin B Vitamin C Vitamin E Vitamin A Calcium Fe Collagen/Royal jelly Turmeric Nourishment Other |
| **31** |  | How often do you take foods for specified health uses per week? | Rarely Almost every day 3-5 days a week 1-2 days a week |
| **32** |  | For those who take foods for specified health uses: What are the effects which you expect? (Multiple answers allowed) | Probiotics Blood pressure Cholesterol Blood glucose level TG Mineral supplementation Tooth Bone Other |
| **33** |  | Have you ever changed your lifestyle, such as diet, exercise, to lead more healthy life? | No Yes |
| **34** |  | For those who answered yes: Please specify the reason that led you make this change? (Multiple choice allowed) | Illness of mine Illness of family Something in test results Diet/Working out -a desire to improve own health Marriage/Career change -Environmental change Partner/Children Preference change Other |
| **35** |  | What are your favorite sports? (Multiple choices allowed) | Sprint Marathon training Ball Game-Individual Ball Game-Team Martial arts Scoring performance Hitting a target Various |
| **36** |  | Can you relieve your fatigue by taking rest? | No Yes |
| **37** |  | How long does your fatigue last? | One time < 1 week < 2 weeks < 4 weeks < 3 months < 6 months >= 6 months |
| **38** |  | Which do you feel tiredness more? At the office/school or at home? | Neither Office/School Home Both |
| **39** |  | Is there anything you do /keep in mind to recover from your fatigue? | No Yes |
| **40** |  | For those who answered yes: Please specify | Sleep Nourishment/Diet Relaxation Circadian rhythm  Enjoy Hobby/holiday Other |
| **41** |  | Do you have the anxiety that your current fatigue may lead to the future incidence of disease? | No Yes |
| **42** |  | Do you have the habit of taking nap? | No Yes |
| **43** |  | Do you have any problem with your sleep? | No Yes |
| **44** |  | For those who answered Yes: Please specify (Multiple choices allowed) | Trouble in falling asleep Don't sleep well Wake up in early morning Can't recover form fatigue Can't wake up in morning Snore Stop breathing in sleep Feeling hot and itching in hands and feet Twitching Talking in your sleep Bruxism Sleep paralysis Nightmare Other |
| **45** |  | Have you ever had trouble staying awake, when you were not supposed to fall asleep, such as while you are driving, eating or during social activity? | No <3 times a month 1-2 times a week >3 times a week Don't know |
| **46** |  | Was there a time when lack of sleep impacted your daily lifestyle or activities? (Fatigue, quality of your work, level of concentration, memory, Feeling, Sleepiness, etc.) | No Sometimes Often Always Don't know |
| **47** |  | Did you take OTC or prescribed drug to help you sleep? | No <3 times a month 1-2 times a week >3 times a week Don't know |
| **48** |  | Are you living by yourself? | No Yes |
| **49** |  | For those who answered No: Who do you live with? (Multiple answers allowed) | Partner Children/ Grandchildren Parents/Grandparents Friends/Housemate Pet Other |
| **50** |  | Do you lead life with regular routines? | No Yes |
| **51** |  | How frequent do you use bathtub when you take shower? | Almost every day 2-3 times a week < once a week |
| **52** |  | What is the temperature of your bath? | Hot Warm Lukewarm |
| **53** |  | Do you dye your hair? | No Yes |
| **54** |  | Which is your dominant hand? | Right Left |
| **55** |  | Do you have bowel movement every day? | >2 times every day once every day 5-6 times a week 3-4 times a week <2 times a week |
| **56** |  | What is your normal condition of your feces? | Diarrhea Loose stool Standard Hard Alternate between constipation and diarrhea |
| **57** |  | Do you have armpit odor? | No Yes |
| **58** |  | What are the things you feel that you got a good deal? (Multiple answers allowed | ○％OFF ○％Increase in volume Points ×　times Buy 〇 and get 〇 free  Free shipping Discount coupon This message is only for you |
| **59** |  | Do you think that the stress levels you experience on daily basis is high? | Very high High Standard Low Very low |
| **60** |  | Did you often feel low or melancholy for past 1 month? | No Yes |
| **61** |  | Was there a time when you continuously feel low or melancholy for over 1 month? | No Yes |
| **62** |  | Did you often feel uninterested in the things or could not really enjoy the things for the past 1 month? | No Yes |
| **63** |  | Was there a time where you could not really interest in or enjoy the things for over 1-month period? | No Yes |
| **64** |  | Do you have any hobby? | No Yes Many hobbies |
| **65** |  | Do you enjoy your daily life? | No Yes Not sure |
| **66** |  | Do you have anybody who make you feel safe and calm down when you meet him/her? | No Yes |
| **67** |  | How many friends do you have whom you talk to more than once /week? | 0 1-3 >4 |
| **68** |  | Do you have anybody who agrees with your action/idea and support? | No Yes |
| **69** |  | Do you have anybody you can share your secret or personal feeling? | No Yes |
| **70** |  | Is your lover opposite sex? | No Yes Both Not applicable |
| **71** |  | Please choose the subject you like most | National language English World history Japanese history  Geography Civics Mathematics Physics Chemistry Biology Earth science Physical education Music Art Technologies / Home economics Information |
| **72** |  | Please choose the subject you are most good at | National language English World history Japanese history  Geography Civics Mathematics Physics Chemistry Biology Earth science Physical education Music Art Technologies / Home economics Information |
| **73** |  | Did you take any lessons when you were a child? (Multiple answers allowed) | Private tutoring school Private tutoring school (for an entrance exam) Language school Exercise Music Art Other |
| **74** |  | What kind of lessons does your child take? (Or you would like to have your child to take in the future?)  Multiple answers allowed | Private tutoring school Private tutoring school (for an entrance exam) Language school Exercise Music Art Other |
| **75** |  | Have you ever married? | No Yes >2 times |
| **76** |  | For those who married: Have you ever experienced divorce? | No Yes >2 times |
| **77** |  | What is the best way for you to memorize the things? | Visual stimulation Hearing stimulation Touching stimulation Linguistic stimulation Other |
| **78** |  | When was the time you were most "popular" (i.e. popular among the opposite sex) Multiple answers allowed | Before elementary school elementary school Junior high school High school University/college/Junior college Graduate school Early 20's Late 20's 30's 40's >40's |
| **79** |  | Is your style of having a relationship "Aggressive" or " Passive" ("Aggressive"=Say "I love you" from your side: "Passive" wait for the approach taken by the counterpart) | Always aggressive Aggressive Passive Always passive Neutral Never fall in love |
| **80** |  | Do you think that there is a "Soul mate”? | No Not for me, but for someone not sure Maybe Must be |
| **81** |  | What is the aspect you think is important when you choose your partner? | Face  Style  Character Economic strength Sexual compatibility Values/Common sense Educational background Political belief/Religion Narcissism Pedigree/Property Healthy Hobby/Sense Compatibility with parents Other |
| **82** |  | Have you measured your blood pressure at health checkups, etc.in the past year? | No Yes |
| **83** |  | Have you measured your total cholesterol value at health checkups, etc. in the past year? | No Yes |
| **84** |  | Have you ever measured your Triglyceride level at health checkups, etc. in the past year? | No Yes |
| **85** |  | Have you ever measured your Fasting Blood sugar level in the past year? | No Yes |
| **86** |  | Please answer if is there any item listed in the above whose score exceeded the standard value at  the health checkup within the past year | Red Blood Cell（RBC） White Blood Cell（WBC） Hemoglobin（Hb） Hematocritト（Hit） Platelet Total cholesterol HDL Cholesterol Triglyceride (TG) LDL Cholesterol GOT（AST） GPT（ALT） γ-GTP ALP ZTT LDH Total bilirubin A/G　ratio Total Protein (TP) Serum Amylase（AMY） Urea Nitrogen（UN） Creatinine（CRE） Uric Acid（UA） HbA1c（NGSP） HBs antigen/Antibody TPHA・RPR RA CRP |
| **87** |  | Are you taking medication on regular basis? | No Yes |
| **88** |  | For those who answered Yes: Please specify the type of drug you are taking (Multiple choices allowed | Treating Hypertensives Cholesterol reducer Diabetes Gout Depression Others |
| **89** |  | Have you ever experienced the adverse reaction caused by the medication you took? | No Yes |
| **90** |  | Is the OTC painkiller effective for your physical makeup? | No Yes It depends on drugs Don't know/Never drink |
| **91** |  | Are you aware of any of the following symptoms for the past one year? |  |
|  | 1 | Squeezing chest pain, or chest pain that continues longer than 10 seconds | No Yes |
|  | 2 | Abnormal heartbeat or being diagnosed to have the arrhythmia | No Yes |
|  | 3 | Suddenly feel as if your tongue is tangled | No Yes |
|  | 4 | Feel as if your hands or feet crippled or paralyzed | No Yes |
|  | 5 | When you walk, start feeling numb or pain after a while, though you were fine before you start walking,  which you can recover after taking a rest | No Yes |
| **92** |  | Have you ever taken Female hormonal agent? | No Yes |
| **93** |  | Have you ever gotten pregnant? | No Yes |
| **94** |  | For those who gave birth: Have you ever breastfed | No Yes |
| **95** |  | Have you ever caught the following disease? | Mastopathy Endometritis Uterine fibroids Ovarian cyst Other |
| **96** |  | Has any of your blood relatives such as your mother, daughter or sisters caught the following diseases? （Multiple choices allowed）※For those who are still alive, please fill in the information being agreed to be shared. For those who are diseased, please fill in the information as far as know | Mastopathy Endometritis Uterine fibroids Ovarian cyst Other |
| **97** |  | What is your bust size? | A B C D E F G >H Don't want answer |
| **98** |  | Have you ever experienced rough skin caused by cosmetics? | No Yes |
| **99** |  | What is your blood type? | A B O AB Unknown |
| **100** |  | Do you have any concern over the followings? (Multiple choices allowed) | Thin hair Body odor Erectile dysfunction Frequent urination Other |
| **101** |  | For those who answered "Thinning hair”: What is your type? (Multiple answers allowed) | Forehead M-shaped Forehead A-shaped Back of the head O-shaped Alopecia areata Overall Thin hair |
| **102** |  | For those who answered "Body Odor": Which odor of the followings do you have concern? (Multiple answers allowed) | Sweat Armpit Foot Mouse Aging body odor Other |
| **103** |  | For those who answered ED: Please specify, if any, the possible cause (Multiple answers allowed) | Chronic disease -Diabetes etc. Psychogenic-Depression, Stress External wound Adverse effect-hypotensive drug etc. Pressure- Honeymoon etc. Other |
| **104** |  | What is your blood type? | A B O AB Unknown |
| **105** |  | Did you have a history of this? |  |
|  | 1 | Cancer | Esophageal Cancer Gastric Cancer CRC Liver Cancer Pancreatic Cancer Bile duct Cancer Kidney Cancer Blood cancer Familial Adenomatous Polyposis Peutz-Jeghers Syndrome Lung Cancer Malignant Lymphoma Pharyngeal and Laryngeal Cancer Thyroid Gland Cancer Bone Tumor Skin Cancer Prostate Cancer Testicular Tumor Breast Carcinoma Cervical Cancer Uterine Body Cancer Ovarian Cancer Uterine Cancer |
|  | 2 | CNS disease | Epilepsy Cerebral Infarction Cerebral Aneurysm Intracerebral Hemorrhage Subarachnoid Hemorrhage Unruptured Cerebral Aneurysm Polyneuritis Peripheral Neuropathy Parkinsonian Syndrome Parkinson's Disease ALS (Amyotrophic Lateral Sclerosis) |
|  | 3 | Respiratory disease | Chronic Obstructive Pulmonary Disease (COPD) Pulmonary Tuberculosis Bronchial Asthma Pneumoconiosis Interstitial Pneumonia Pneumonia Pleurisy Pulmonary Fibrosis Nontuberculous Mycobacteriosis Apnea Syndrome |
|  | 4 | Circulatory organ disease | Myocardial infarction Unstable angina Stable angina Arrhythmia (Ventricular Arrhythmia) Arrhythmia (Atrial fibrillation) Arrhythmia (Details unknown) Heart Failure Valvular Disease Dilated Cardiomyopathy Hypertrophic Cardiomyopathy Myocarditis Pericarditis Hypertension Aortic Aneurysm Arteriosclerosis Obliterans (ASO) |
|  | 5 | Digestive organs disease | Gastric Ulcer Duodenal Ulcer Ulcerative Colitis Crohn's Disease Reflux Esophagitis Hepatitis B Hepatitis C Cirrhosis Cholecystitis Gallstone Acute Pancreatitis Chronic Pancreatitis |
|  | 6 | Urinary system disease | Nephrotic Syndrome Acute Glomerulonephritis Chronic Glomerulonephritis Acute Renal Failure Chronic Renal Failure Cystic Kidney Kidney Stone Ureteral Calculus Neurogenic Bladder Vesicoureteral Reflux Prostatic Hypertrophy |
|  | 7 | Hematology disease | Anemia (iron deficiency) Anemia (defective regeneration) Idiopathic Thrombocytopenic Purpura Acute Myeloid Leukemia Chronic Myelogenous Leukemia Acute Lymphocytic Leukemia Chronic Lymphocytic Leukemia Multiple Myeloma |
|  | 8 | Metabolism disease | Type 1 Diabetes Type 2 Diabetes Familial Juvenile Diabetes (MODY) Mitochondrial Diabetes Diabetes Mellitus (Details unknown) Hyperlipidemia Osteoporosis Hyperuricemia and gout Amyloidosis |
|  | 9 | Endocrine disease | Graves' disease Hashimoto's disease Hypothyroidism Hyperthyroidism Goiter Pheochromocytoma Acromegaly Cushing's syndrome Cushing's disease |
|  | 10 | Collagen disease | Rheumatoid Arthritis Juvenile Rheumatoid Arthritis Malignant Rheumatoid Arthritis Systemic Lupus Erythematosus Sjogren’s Syndrome Progressive Systemic Sclerosis Polymyositis Dermatomyositis |
|  | 11 | Allergy | Pollinosis Allergic conjunctivitis Allergic rhinitis Vernal catarrh Food allergy Atopic dermatitis Hives Drug Eruption (toxic epidermal necrolysis) Drug Eruption (Stevens-Johnson syndrome) Drug Eruption (Details unknown) |
|  | 12 | Pediatrics disease | Febrile Convulsion Mental Retardation Motor Development Delay Malformation Infant Respiratory Tract Infection Infant Undernutrition Perinatal Abnormality Attention Deficit Hyperactivity Disorder Autism |
|  | 13 | Ophthalmology disease | Cataract Glaucoma Retinal Detachment Episcleritis Iritis Retinitis Pigmentosa Age-related Macular Degeneration Myopia Astigmatism |
|  | 14 | Face/Head disease | Chronic otitis Media Chronic Sinusitis Hearing Loss Meniere's Disease Periodontal Disease Light Sneezing Reflex Migraine Headache Cluster Headache Alopecia Areata Male-pattern Alopecia (AGA) |
|  | 15 | Psychiatry disease | Depression Insomnia Anorexia Bulimia Addiction (Shopping, alcohol, drugs, people) Narcolepsy Alzheimer's Schizophrenia Manic-depressive Psychosis |
|  | 16 | Infectious disease and others | HIV Influenza Rubella Measles Tooth Decay Malaria Parasite Food Poisoning Venereal Disease |
| **106** |  | In your average week, how many days you do Heavy(Vigorous intensity) physical activities （Carry heavy luggage, climb the hill by bicycle, jogging,　Tennis singles match） | No 1 day 2 days 3 days 4 days 5 days 6 days Everyday |
| **107** |  | In your average week, how many days you do the moderate physical activities? (Carry light weight luggage, play tag with children, swim slowly, Tennis doubles match, playing golf without using cart) Please do not include "walking" | No 1 day 2 days 3 days 4 days 5 days 6 days Everyday |
| **108** |  | In your average week, how many days do you walk continuously over 10 minutes? In this question "walk" includes any walking activities such as in your work/daily life, leisure walking or brisk walking | No 1 day 2 days 3 days 4 days 5 days 6 days Everyday |
| **109** |  | Please fill in the schools you went (multiple answers allowed) | Elementary/Junior high school High school Junior college Technical college Business college University Graduate school Student Other（Prep school etc. |
| **110** |  | Have you smoked more than 100 tobacco in total, since you were born? | No Yes |
| **111** |  | Are you allergic to substances other than food/medications? | No Yes |
| **112** |  | For those who answered yes: Do you know the substance causing your allergy (multiple answers allowed) | Tick Dandruff and fur of pets Fungus/Mold Insects Other / house dust Pollen Other |
| **113** |  | Did you take health checkup for the past year? | No Yes |
| **114** |  | If not, what was the reason (multiple answers allowed) | Don't know Don't have time Far from home Costs Anxious about test While hospitalization Don't think annual testing is necessary Don't think it is necessary for healthy people Can see a doctor whenever I don't feel well Don't want to see a test result Bothering Other |
| **115** |  | Did you measure HbA1c for the past year at health checkups etc. | No Yes |
| **116** |  | How often do you take antipyretic/pain reducer? | Don't take <once a half year 2-5 times a half year 1-3 times a month once /twice a week >3 times a week |
| **117** |  | Have you ever received blood transfusion or bone marrow transplantation? | No Only blood transfusion Only bone marrow transplant Both |
| **118** |  | Please answer the questions about your main job (The followings will be used for behavioral economic. We ask you for your cooperation) | Unemployed Management Technical staff Admin assistant Sales Service Security Agriculture, forestry and fisheries Transport / communication Production process/labor work homemaker Other |
| **119** |  | What is the employment status of your current work? | Full-time employee Contract employee Temporary employee Part time job Manager Not applicable |
| **120** |  | Except for those who the above question is not applicable for: Please choose from options about the number of employees. | 1(myself) 2-5 6-20 21-100 101-300 >301 |
| **121** |  | Do you make it a habit to save money? | No Yes |
| **122** |  | Do you have the asset? (multiple answers allowed) | Deposit Securities Insurance Real estate Luxury car/Jewelry Other Don't want answer |
| **123** |  | Please choose from the answers most like your current situation of your household | Barely enough to make living, cannot pay for the other things Not suffer from hunger but cannot buy everything I want Can buy almost everything I want Can afford to do many things |
| **124** | 1 | Had you vaccinated for flu before 2008? | No Yes Don't remember |
|  | 2 | Have you ever vaccinated for flue after 2009? | No Yes Don't remember |
|  | 3 | Had you ever experienced flu-like symptoms before 2008? （Cold, joint pain, muscle aches, cough associated with fever 38 ℃　and higher ） | No Yes Don't remember |
|  | 4 | For those who selected "Yes": How many times did you visit hospital when you experienced the above symptoms, and how many times diagnosed with flu | No 1 time 2 times >3 times Don't remember |
| **125** |  | For those who were diagnosed with flu before 2008　 Had you vaccinated for flu at the medical institutions in the year you were diagnosed with flu ? Please choose "Yes " if you had the vaccine 1time and more | No Yes（Injection） Yes（Nasal drop） Don't remember |
| **126** |  | Had you ever been hospitalized before 2008 due to flu? | No Yes |
| **127** |  | Has any of your blood relatives vaccinated for flu before 2008? | No Yes（Partially） Yes（All） Don't remember |
| **128** |  | Had you ever experienced flu-like symptoms after 2009? （Cold, joint pain, muscle aches, cough associated with fever 38 ℃　and higher ） | No Yes Don't remember |
| **129** |  | For those who selected "Yes": Did you visit hospital when you experienced the above symptoms? How many times had you been diagnosed with flu? | No 1 time 2 times >3 times Don't remember |
| **130** |  | For those who were diagnosed with flu before 2009　 Had you vaccinated for flu at the medical institutions in the year you were diagnosed with flu ? Please choose "Yes " if you had the vaccine 1time and more | No Yes（Injection） Yes（Nasal drop） Don't remember |
| **131** |  | Have you ever been hospitalized due to flu after 2009? | No Yes |
| **132** |  | Has any of your blood relatives vaccinated for flu after 2009? | No Yes（Partially） Yes（All） Don't remember |
| **133** |  | What is the area, if any, you would like to improve your life? (Multiple answers allowed) | Exercise Diet Sleep Work Hobby/Rest Stress/Fatigue Other |
| **134** |  | What was the frequency you have experienced the feelings listed below? |  |
|  | 1 | Did you feel nervous? | Never Rarely Sometimes Often Always |
|  | 2 | Did you feel hopeless? | Never Rarely Sometimes Often Always |
|  | 3 | Were you restless? | Never Rarely Sometimes Often Always |
|  | 4 | Did you feel depressed and felt like nothing could cheer you up? | Never Rarely Sometimes Often Always |
|  | 5 | Did you feel tired to do anything? | Never Rarely Sometimes Often Always |
|  | 6 | Did you feel you were a worthless person? | Never Rarely Sometimes Often Always |

**Supplementary Table 2.** QC Table

|  | **HumanCore-12 Custom BeadChip v1.0** | | | | | | **HumanCore-24 Custom BeadChip v1.2** | | | | | | **v1.0+ v1.2 = vM** | | | | | |
| --- | --- | --- | --- | --- | --- | --- | --- | --- | --- | --- | --- | --- | --- | --- | --- | --- | --- | --- |
|  | **Subjects** | **diff** | **%** | **Variants** | **diff** | **%** | **Subjects** | **diff** | **%** | **Variants** | **diff** | **%** | **Subjects** | **diff** | **%** | **Variants** | **diff** | **%** |
| Remvoe subjects with inconsistent sex | 11025 | 18 | 0.16 | 302072 | 0 | 0 | 1583 | 9 | 0.57 | 309725 | 0 | 0.00 |  |  |  |  |  |  |
| Remove variants on sex chromosome | 11025 | 0 | 0.00 | 291786 | 10286 | 3.41 | 1583 | 0 | 0.00 | 298454 | 11271 | 3.64 | 12596 | 0 | 0 | 230375 | 0 | 0.00 |
| Remove variants (call rate < 0.99) | 11025 | 0 | 0.00 | 264051 | 27735 | 9.51 | 1583 | 0 | 0.00 | 244008 | 54446 | 18.24 | 12596 | 0 | 0 | 230375 | 0 | 0.00 |
| Remove subjects (call rate < 0.99) | 11024 | 1 | 0.01 | 264051 | 0 | 0 | 1572 | 11 | 0.69 | 244008 | 0 | 0.00 | 12596 | 0 | 0 | 230375 | 0 | 0.00 |
| Remove variants (HWE P < 1e-6) | 11024 | 0 | 0.00 | 263975 | 76 | 0.03 | 1572 | 0 | 0.00 | 243964 | 44 | 0.02 | 12596 | 0 | 0 | 230334 | 41 | 0.02 |
| Remove variants (MAF < 0.01) | 11024 | 0 | 0.00 | 219238 | 44737 | 16.9 | 1572 | 0 | 0.00 | 201833 | 42131 | 17.27 | 12596 | 0 | 0 | 189753 | 40581 | 17.62 |
| Remove non-East Asian subjects |  |  |  |  |  |  |  |  |  |  |  |  | 12586 | 10 | 0.08 | 189753 | 0 | 0.00 |
| Remove non-Japanese subjects |  |  |  |  |  |  |  |  |  |  |  |  | 12522 | 64 | 0.51 | 189753 | 0 | 0.00 |
| Remove either of close relatives (PI_HAT > 0.1875) |  |  |  |  |  |  |  |  |  |  |  |  | 12385 | 137 | 1.09 | 189753 | 0 | 0.00 |

**Supplementary Table 3.** Alcohol sensitivity definition

(a) alcohol intake frequency

| **Response** | **Population** | **Classification** | **Total** |
| --- | --- | --- | --- |
| **Every day** | 2,079 | Control | 9,995 |
| **5-6 days a week** | 1,059 |  |  |
| **3-4 days a week** | 1,142 |  |  |
| **1-2 days a week** | 1,582 |  |  |
| **1-3 days a month** | 1,978 |  |  |
| **Less than the above** | 2,155 |  |  |
| **Never** | 2,638 | Case | 2,638 |

(b) alcohol flush reaction

| **Answer** | **Population** | **Classification** | **Total** |
| --- | --- | --- | --- |
| **Turns Red** | 4,638 | Case | 6,713 |
| **If anything, turns red** | 2,075 |  |  |
| **Does not turn red** | 5,606 | Control | 5,606 |

**Supplementary Table 4.** PheWAS case and control definition template

| **Variables** | **Categories** | **Choice options** | **Case/Control** |
| --- | --- | --- | --- |
| unordered categorical variables | ex.) apple, orange, grape | Single choice | apple/orange, grape  orange/apple, grape  grape/apple, orange |
|  |  | multiple-choice | Individuals who chose apple/ individuals who did not choose apple  Individuals who chose orange/ individuals who did not choose orange  Individuals who chose grape/ individuals who did not choose grape |
| ordered categorical variables | ex.) a, b, c | Single choice | a/b, c  a, b/c |

Supporting Figures


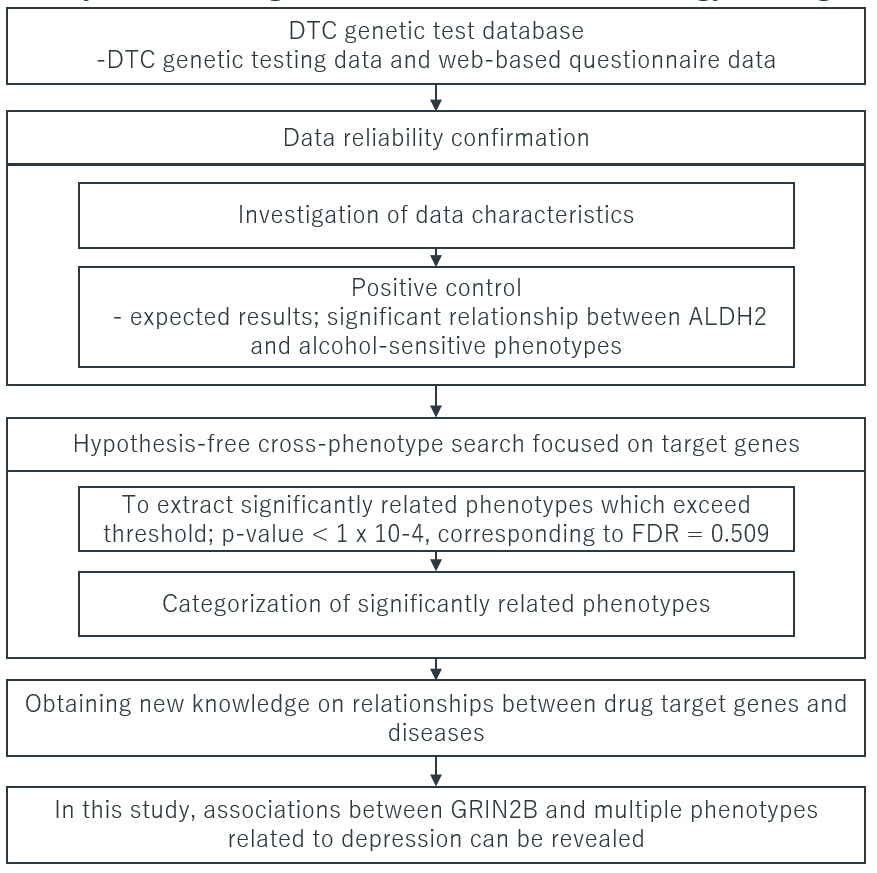


Figure S1. Flow chart of obtaining new knowledge on relationships between drug target genes and diseases using DTC genetic test database


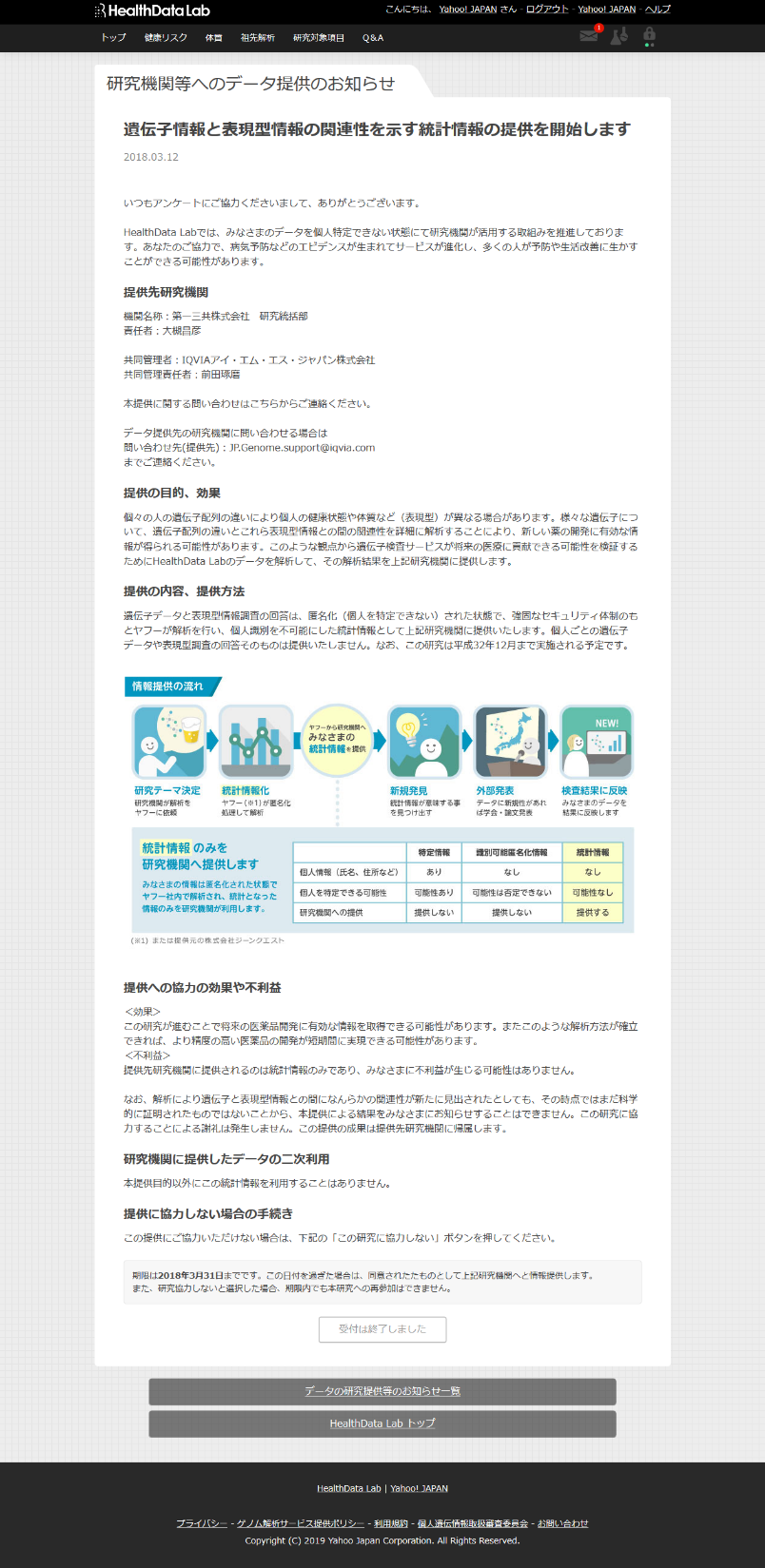


Figure S2. Agreement of data share for research (opt-out form)


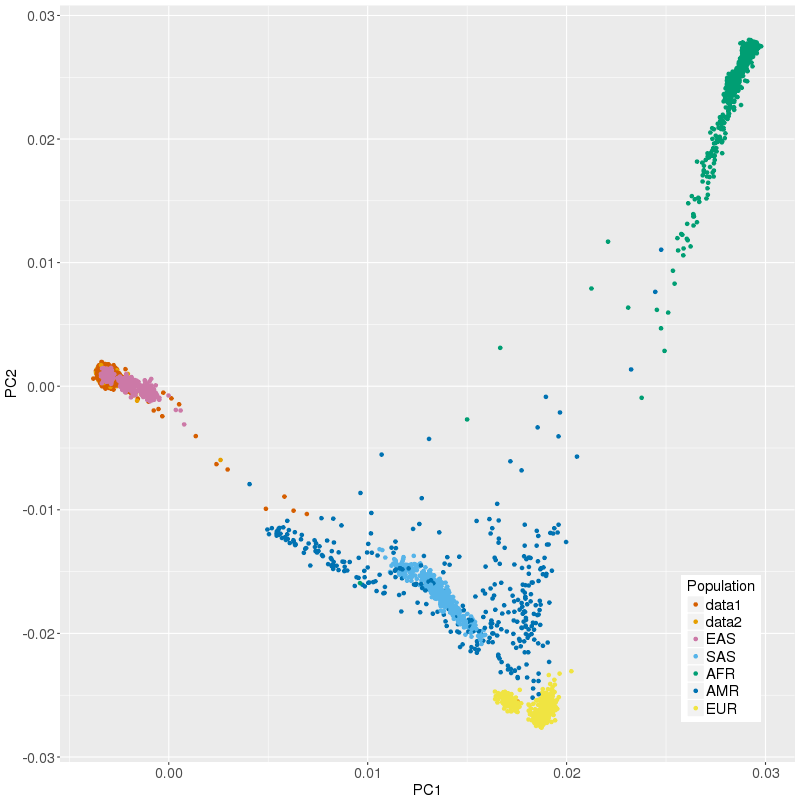

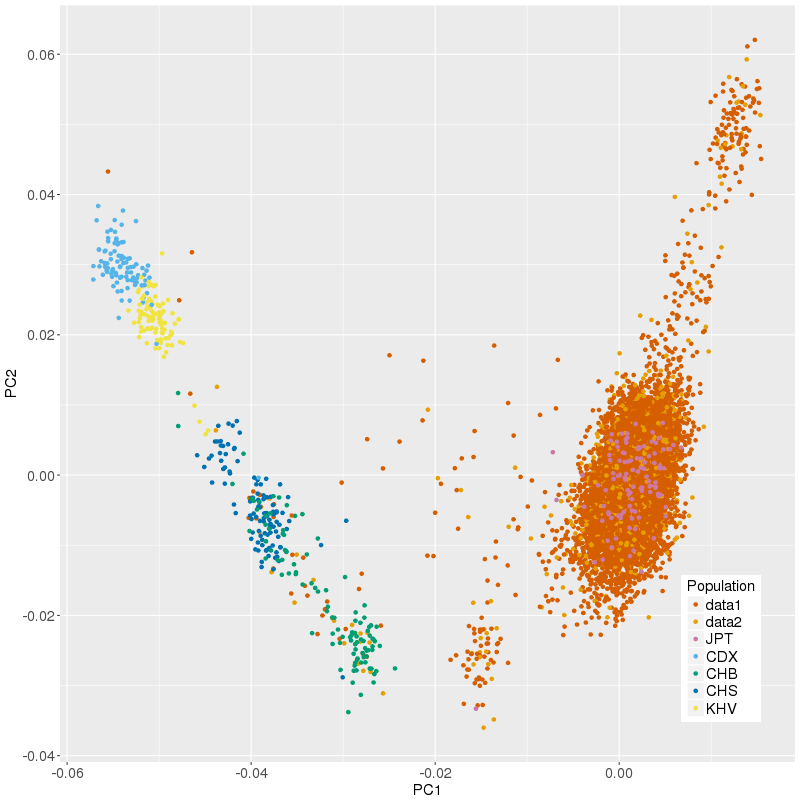


Figure S3. Principal component analysis using data for this study

data1 (HumanCore-12), data2 (HumanCore-24), all samples consisting of EAS(East Asian), SAS (South Asian), AFR　(African), AMR (Ad Mixed American), and EUR (European) in the left figure, and East Asian population samples consisting of JPT (Japanese in Tokyo, Japan), CDX (Chinese Dai in Xishuangbanna, China), CHB (Han Chinese in Beijing, China), CHS (Southern Han Chinese), and KHV (Kinh in Ho Chi Minh City, Vietnam) in the right figure. X-axis and Y-axis indicate PC1 and PC2, respectively.


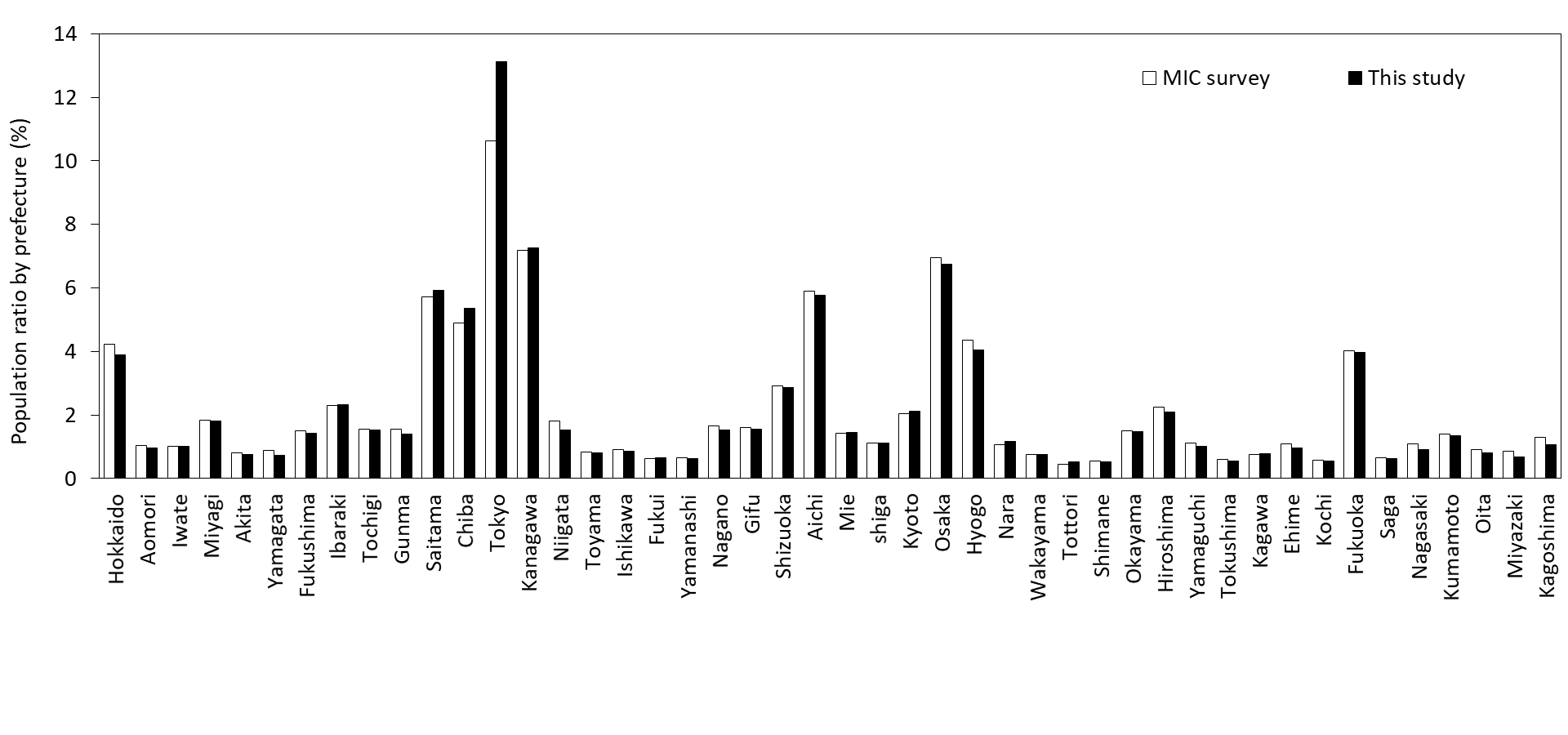


**Figure S4.** The population ratio of subjects among birth prefectures and comparison with Japanese population.

References

1. *Ethical Guidelines for Human Genome / Gene Analysis Research*, in *Ministry of Health, Labor and Welfare*. 2001.

2. Tsutsumi, M., *Outline of revision of "Ethical Guidelines for Human Genome / Gene Analysis Research".* Organ Biology, 2014. **21**(1): p. 9-15.

3. *Personal Genetic Information Handling Review Committee in HealthData Lab*. Available from: <https://health.yahoo.co.jp/service/ethics>. (last visited on Jun 14 2020)

4. Price, A.L., et al., *Principal components analysis corrects for stratification in genome-wide association studies.* Nat Genet, 2006. **38**(8): p. 904-9.

5. Genomes Project, C., et al., *A global reference for human genetic variation.* Nature, 2015. **526**(7571): p. 68-74.

6. Purcell, S., et al., *PLINK: a tool set for whole-genome association and population-based linkage analyses.* Am J Hum Genet, 2007. **81**(3): p. 559-75.

7. Loh, P.R., et al., *Reference-based phasing using the Haplotype Reference Consortium panel.* Nat Genet, 2016. **48**(11): p. 1443-1448.

8. Das, S., et al., *Next-generation genotype imputation service and methods.* Nat Genet, 2016. **48**(10): p. 1284-1287.

9. Yoshida, A., I.Y. Huang, and M. Ikawa, *Molecular abnormality of an inactive aldehyde dehydrogenase variant commonly found in Orientals.* Proc Natl Acad Sci U S A, 1984. **81**(1): p. 258-61.
